# an-QNA: An Adaptive Nesterov Quasi-Newton Acceleration-Optimized CMOS LNA for 65 nm Automotive Radar Applications

**DOI:** 10.3390/s24186141

**Published:** 2024-09-23

**Authors:** Unal Aras, Lee Sun Woo, Tahesin Samira Delwar, Abrar Siddique, Anindya Jana, Yangwon Lee, Jee-Youl Ryu

**Affiliations:** 1 Department of Smart Robot Convergence and Application Engineering, Pukyong National University, Busan 48513, Republic of Korea; unalaras21.20@gmail.com (U.A.); dltjsdn2005@gmail.com (L.S.W.); samira.fset@gmail.com (T.S.D.); abrarkhokhar.iiui@gmail.com (A.S.); 2 Department of Electronics & Communication Engineering, JB Institute of Engineering & Technology, (Autonomous), Hyderabad 500075, India; anindya.jana@rediffmail.com; 3 Department of Spatial Information Engineering, Pukyong National University, Busan 48513, Republic of Korea

**Keywords:** adaptive Nesterov quasi-Newton acceleration (an-QNA), conventional quasi-newton (c-QN), global convergence, highly linear, low-noise amplifier (LNA), improved post-linearization (I_PL_)

## Abstract

An adaptive Nesterov quasi-Newton acceleration (an-QNA)-optimized low-noise amplifier (LNA) is proposed in this paper. An optimized single-ended-to-differential two-stage LNA circuit is presented. It includes an improved post-linearization (I_PL_) technique to enhance the linearity. Traditional methods like conventional quasi-Newton (c-QN) often suffer from slow convergence and the tendency to get trapped in local minima. However, the proposed an-QNA method significantly accelerates the convergence speed. Furthermore, in this paper, modifications have been made to the an-QNA algorithm using a quadratic estimation to guarantee global convergence. The optimized an-QNA-based LNA, using standard 65 nm CMOS technology, achieves a simulated gain of 17.5 dB, a noise figure (NF) of 3.7 dB, and a 1 dB input compression point (IP_1_dB) of −13.1 dBm. It is also noted that the optimized LNA achieves a measured gain of 12.9 dB and an NF of 4.98 dB, and the IP_1_dB is −17.8 dB. The optimized LNA has a chip area of 0.67 mm^2^.

## 1. Introduction

**Figure 1:**
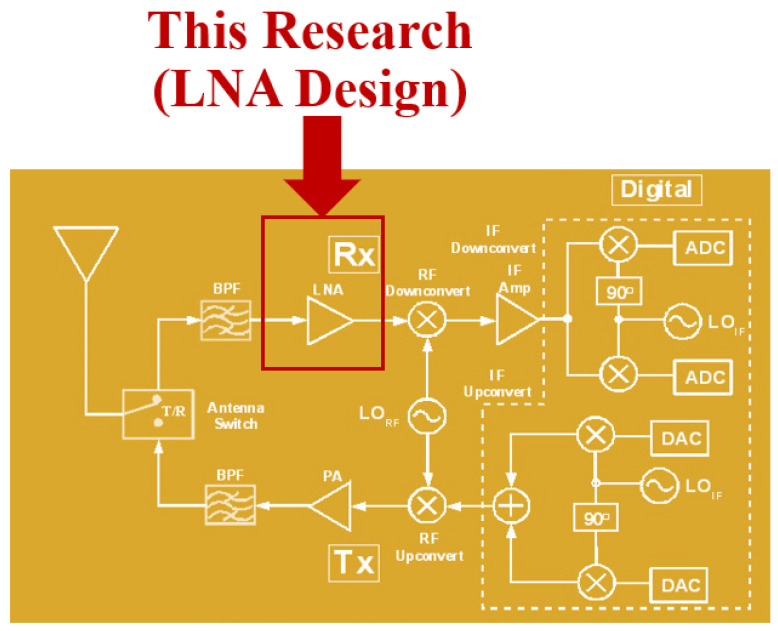
Overview of RF transceiver.

**Figure 2:**
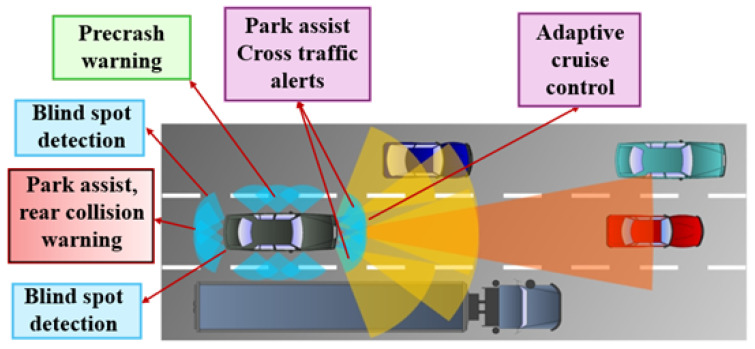
A 24 GHz automotive radar application.

A complementary metal-oxide semiconductor (CMOS) is a fundamental technology used to make integrated circuits (ICs), such as microprocessors and microcontrollers. Over the years, CMOS technology has improved tremendously. A big breakthrough in RF (radio frequency) and mixed-signal IC design came with the combination of low-noise amplifiers (LNAs) and CMOS technology [1]. The LNA is a key component of automotive radar systems. Automotive radars operate primarily at frequencies around 24 GHz [2]. Figure 1 represents the overview of RF transceiver, and Figure 2 represents the radar application.

The challenge, however, remains to design CMOS LNA for automotive radar systems. We are still in the early stages of designing and automating LNA circuits. The design of the LNA becomes more difficult when it works at high frequencies [3]. The high non-linearity of its parameters means that the researchers have a hard time designing a high-performance economic circuit on their own due to non-linearity. Additionally, the design of optimal LNAs for automotive radars is challenging because they must have wide bandwidths, low noise levels, and high linearity in order to pick up weak signals while ignoring strong ones. In order to achieve a delicate balance between these two tenets, intricate design optimizations would be necessary along with multidisciplinary expertise in RF circuitry and multilevel considerations at the system level.

From the prior research, it is evident that optimization algorithms (OAs) [4] play a significant role in the design process. Recent advances in intelligent paradigm research have led to a number of theoretical advancements and to successful applications of intelligent techniques and approaches to a variety of fields [5,6]. In many optimization problems, linear programming [7], Newton’s method [8], gradient search methods [9], quadratic programming, and nonlinear programming [10] are used. The issue of LNA circuits has been addressed in the literature by many optimization techniques, including genetic algorithms (GA) [11], particle swarm optimization (PSO) [12], flower pollination [13], firefly algorithms (FA) [14], ant colony (AC) [15], and artificial bee colony (ABC) [16].

The PSO method was used by Thakker and Fakhfakh et al. [17,18] for the automatic sizing of low-power analog circuits, the automatic synthesis of LNA [19], and the design of microwave filters [20]. It has been shown that a GA can be used to optimize several electrical parameters of LNAs [21]. In [22,23], for analog and RF circuit a highly efficient parasitic aware hybrid sizing methodology is proposed. According to Yiming Li et al. [24] GA generalizes the method for optimizing parameters for LNA circuits with respect to constraints. However, the paper has limitations, as using a small population size could result in unsatisfactory convergence behavior. A recent report [25,26] also noted optimized design parameters for LNAs using PSO. As demonstrated in [27], the iterative loop between the OAs and the performance evaluator allows the multi-objective artificial bee colony (MOABC) algorithm to optimize the voltage gain and noise figure.

In order to accelerate the process of convergence to the global maximum, Khong et al. [28] used direct global optimization techniques to implement a multi-unit algorithm. According to Shams et al. [29], several parameters of the LNA were optimized using GSA, including S_21_, S_11_, S_22_, S_12_, and the noise figure (NF). GSA’s clustered GSA (CGSA) has been successfully exploited to reduce computational complexity, improve efficiency, and improve robustness. In [30,31], genetic computation was utilized to circumvent parasitics and achieve optimal results, but no mathematical analysis was provided. A cascade LNA was designed and optimized using PSO [32].

As part of our work, we chose adaptive Nesterov quasi-Newton acceleration (an-QNA) to optimize LNA at 24 GHz. An-QNA benefits high-frequency applications because it can be converged and optimized more quickly. With an-QNA, optimization processes are smoother, and convergence is faster. Evolutionary algorithms (EAs), however, may be preferred when the optimization is highly non-linear and non-convex or when the exploration of unconventional design spaces is required, though they may be slower to reach convergence. In contrast to conventional quasi-Newton (c-QN), which usually struggles with slow convergence and gets stuck in local minima, an-QNA guarantees faster global convergence. We have made modifications to the an-QNA algorithm to guarantee global convergence. Therefore, in the fair comparison in our work, we compare c-QN with the proposed an-QNA to optimize the 24 GHz LNA.

### Main Contributions

In the following list, we summarize and discuss the paper’s main contributions:

1. The paper presents an an-QNA designed to improve the performance of LNAs. The proposed an-QNA significantly accelerates the convergence speed compared to c-QN, which often suffer from slow convergence and local minima issues.

2. The proposed LNA circuit includes an improved post-linearization (I_PL_) technique. This technique enhances the linearity of the LNA, a critical factor in achieving high-performance automotive radar applications.

3. In the proposed an-QNA, we have made modifications using a quadratic estimation to the an-QNA algorithm to ensure global convergence, addressing a significant challenge in OAs used in circuit design.

4. Utilizing standard 65 nm CMOS technology, the optimized LNA achieves a simulated gain of 17.5 dB and an NF of 3.7 dB as well as a 1 dB input compression point (IP_1_dB) of −13.1 dBm. Furthermore, the optimized LNA achieves a measured gain of 12.9 dB and an NF of 4.98 dB in addition to an IP_1_dB of −17.8 dB.

This paper consists of four sections. The proposed LNA circuit using an-QNA is described in Section 2. The simulation results are presented in Section 3, and in Section 4, the conclusions and future research directions are given.

## 2. Proposed Methodology for Optimizing an LNA Circuit

### 2.1. Adaptive Nesterov Quasi-Newton Acceleration (an-QNA)

an-QNA is an OA algorithm that combines the concepts of Nesterov’s acceleration with quasi-Newton methods while introducing adaptivity to improve convergence speed and efficiency. c-QN methods are a class of OAs used to find the minimum (or maximum) of a function. In comparison to c-QN, the an-QNA convergence process is much faster.

### 2.2. Modifications in an-QNA

To achieve a faster convergence in an-QNA, we included a quadratic estimation at w_i_ + μz_i_. Additionally, we added Nesterov’s enhanced gradient, namely ∇F (w_i_ + μz_i_). The an-QNA modification is briefly outlined as follows. In our work, we consider Equations (1)–(3),

(1) $$F\left(w\right)=\frac{1}{\left|T_{r}\right|}\sum_{p\epsilon \left(T_{r}\right)}F_{p\left(w\right)}$$

(2) $$F_{p}\left(w\right)=\frac{1}{2}{∥d_{p}-o_{p}∥}^{2}$$

(3) $$F\left(w\right)≃F\left(w_{i}+\mu_{i}z_{i}\right)+\nabla F{\left(w_{i}+\mu_{i}\right)}^{T}\nabla w+\frac{1}{2}\nabla w^{T}\nabla^{2}F\left(w_{i}+\mu_{i}z_{i}\nabla w\right)$$

There is a precise minimization of this quadratic function in Equation (4),

(4) $$\nabla w=-\nabla^{2}F{\left(w_{i}+\mu_{i}z_{i}\right)}^{-1}\nabla F\left(w_{i}+\mu_{i}z_{i}\right)$$

It is considered as Newton’s method with momentum added μz_i_ in Equation (5).

(5) $$w_{i+1}=\left(w_{i}+\mu_{i}z_{i}\right)-\nabla^{2}F{\left(w_{i}+\mu_{i}z_{i}\right)}^{-1}\nabla F\left(w_{i}+\mu_{i}z_{i}\right)$$

The inverse of Hessian in Equation (6), ∇F (w_i_ + μ_i_z_i_) is estimated by the matrix H_i+1_,

(6) $${\hat{H}}_{i+1}=\left(\left(I-\hat{\rho_{i}}s_{i}r_{i}^{T}\right){\hat{H}}_{i}\left(I-\rho_{i}s_{i}r_{i}^{T}\right)+\rho_{i}s_{i}r_{i}^{T}\right)$$

where

s_i_ = w_i+1_ − (w_i_ + μ_i_z_i_)

r_i_ = ∇F(w_i+1_)−∇F(w_i_ + μ_i_z_i_),

Then, the renew vector of an-QNA can be written as in Equations (7) and (8),

(7) $$z_{i+1}=\mu_{i}z_{i}-\alpha_{i}{\hat{g}}_{i}$$

(8) $$\hat{g_{i}}=-{\hat{H}}_{i}\nabla F\left(w_{i}z_{i}\right)$$

Thus, the vector in the modified method is given as in Equations (9) and (10),

(9) $${\hat{\xi }}_{i}=w\left\|\nabla F\left(w_{i}+\mu z_{i}\right)\right\|+max\left\{\frac{-\epsilon_{i}^{T}s_{i}}{\|s_{i}{\|}^{2},0}\right\}$$

(10) $$\left\{\begin{array}{cc} \omega =2 & if\;\|\nabla F\left(w_{i}+\mu z_{i}{\|}^{2}>10^{-2}\right)\\ \omega =100 & if\;\|\nabla F\left(w_{i}+\mu z_{i}{\|}^{2}<10^{-2}\right) \end{array}\right\}$$

**Figure 3:**
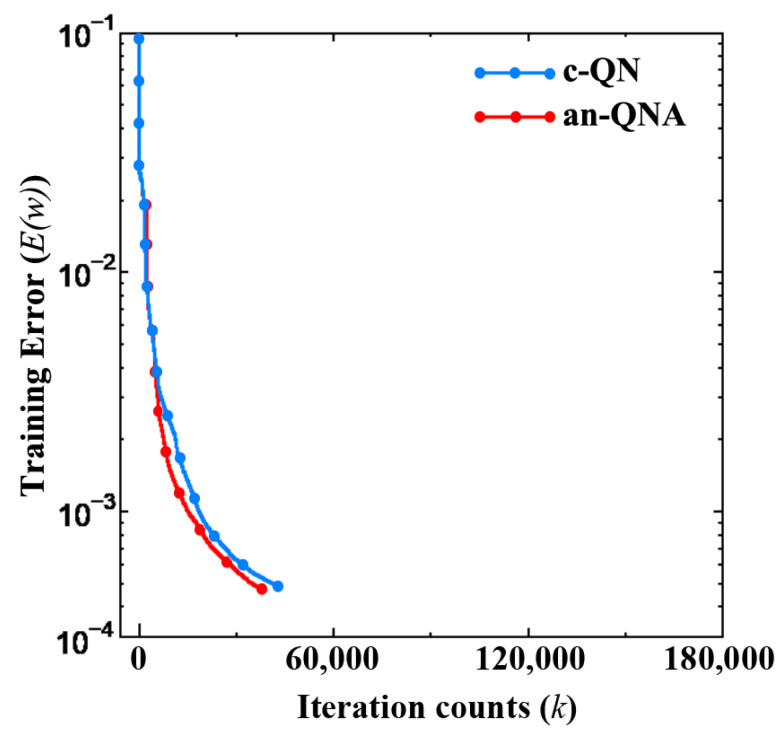
The training error vs. iteration counts of LNA.

**Figure 4:**
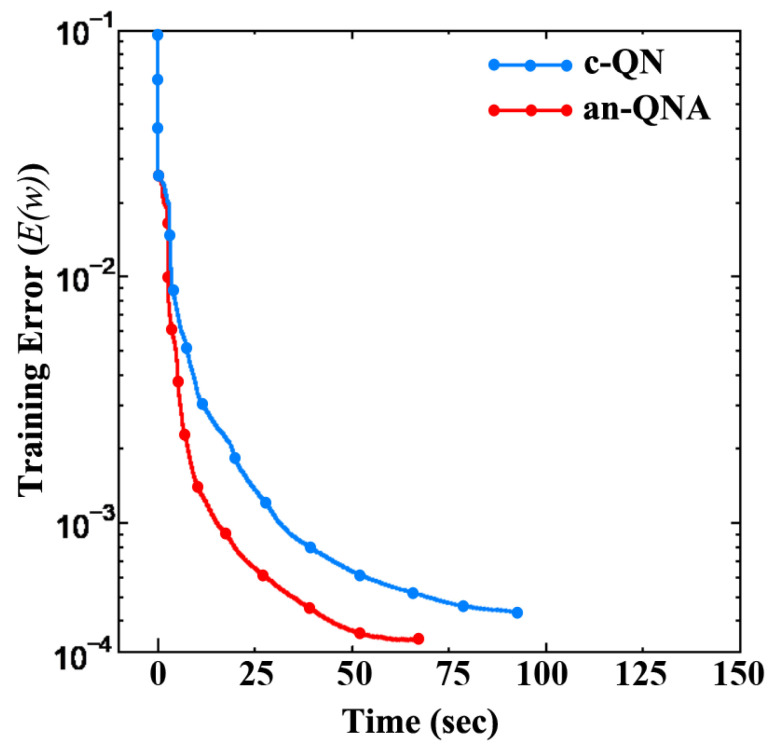
The training error vs. time of LNA.

Figure 3 presents the training error vs. iteration counts of LNA, while Figure 4 shows LNA training error vs. time. Table 1 presents the summary of the simulation results of LNA modeling. From Table 1, we can see that the convergence rate for c-QN is 75% while the convergence rate of the proposed an-QNA is 100%. On the other hand, the c-QN’s execution time is 65 s, and the proposed an-QNA’s execution time is 95 s.

### 2.3. Convergence Performance Analysis of an-QNA

**Figure 5:**
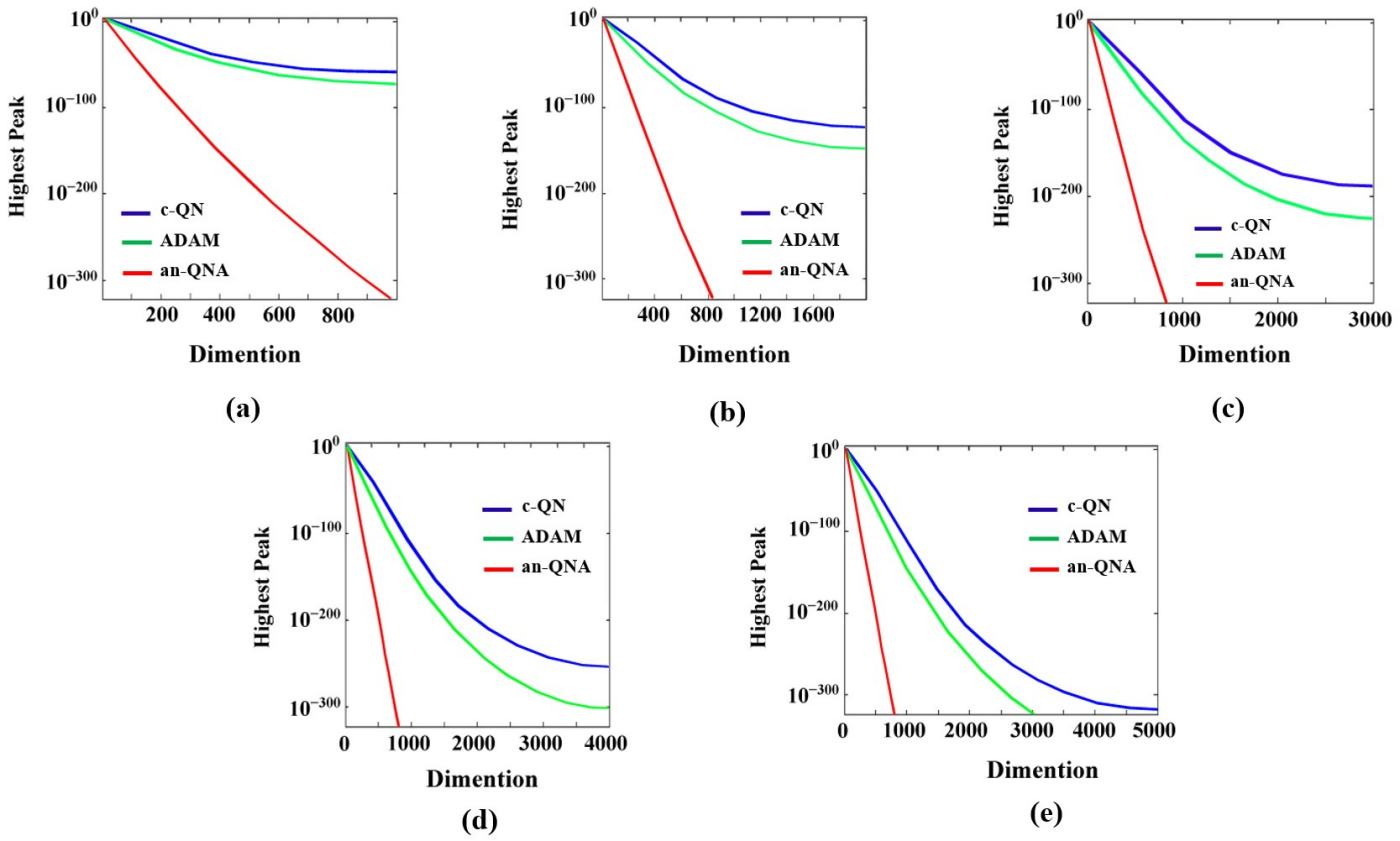
Convergence graphs of c-QN, ADAM, an-QNA algorithms (**a**) 1000 dimension (**b**) 2000 dimension (**c**) 3000 dimension (**d**) 4000 dimension (**e**) 5000 dimension.

In this section, Equations (11)–(18) represent eight benchmark functions, both unimodal and multimodal, which are employed to assess the performance of the an-QNA algorithm. Table 2, provides detailed information regarding the dimensions, minimum range, and function characteristics of these benchmark functions. The performance of gradient-based OAs such as c-QN and adaptive moment estimation (ADAM) has been evaluated alongside the an-QNA variant to examine aspects like convergence rate, stability, and computational accuracy across multiple iterations, as depicted in Figure 5. The results demonstrate that, when comparing the optimal function values plotted over several generations in Figure 5, the an-QNA variant outperforms others in terms of convergence, particularly in scenarios with models of varying sizes, ranging from 1000 to 5000 dimensions. The graphs indicate that for the an-QNA variants, the standard test function values decrease rapidly as the number of generations increases, in contrast to other OAs. In Figure 5, it can be observed that both c-QN and ADAM exhibit slow convergence and tend to stagnate during the partitioning process. However, the an-QNA variant, particularly in terms of the mean strategy, effectively prevents the algorithm from getting trapped in local minima and enhances the search process by speeding up convergence.

(11) $$Function_{v1-unimodal}\left(y\right)=\sum_{i=1}^{n}y_{i}^{2}$$

(12) $$Function_{v1-unimodal}\left(y\right)=\sum_{i=1}^{n}{\left(\sum_{j-1}^{n}y_{j}\right)}^{2}$$

(13) $$Function_{v3-unimodal}\left(y\right)=\sum_{i=1}^{n}\left[{\left(y_{i}+0.5\right)}^{2}\right]$$

(14) $$Function_{v4-unimodal}\left(y\right)=\sum_{i=1}^{n}iy_{i}^{4}+rand\left[0,1\right]$$

(15) $$Function_{v5-multimodal}\left(y\right)=\sum_{i=1}^{n}-y_{i}sin\left(\sqrt{|y_{i}|}\right)$$

(16) $$Function_{v6-multimodal}\left(y\right)=-20exp\left(-0.2\sqrt{\frac{1}{n}\sum_{i=1}^{n}y_{i}^{2}}-exp\left(\frac{1}{n}\sum_{i=1}^{n}cos\left(2\pi y_{i}\right)\right)+20+e\right)$$

(17) $$Function_{v7-multimodal}\left(y\right)=\sum_{i=1}^{n}\left[y_{i}^{2}-10cos\left(2\pi y_{i}\right)\right]+10$$

(18) $$Function_{v8-multimodal}\left(y\right)=\sum_{i=1}^{n}\left[y_{i}^{2}-10cos\left(2\pi y_{i}\right)\right]+10$$

(19) $$Function_{v8-multimodal}\left(y\right)=\frac{1}{4000}\sum_{i=1}^{n}-\prod_{i=1}^{n}cos\left(\frac{y_{i}}{\sqrt{i}}+1\right)$$

**Figure 6:**
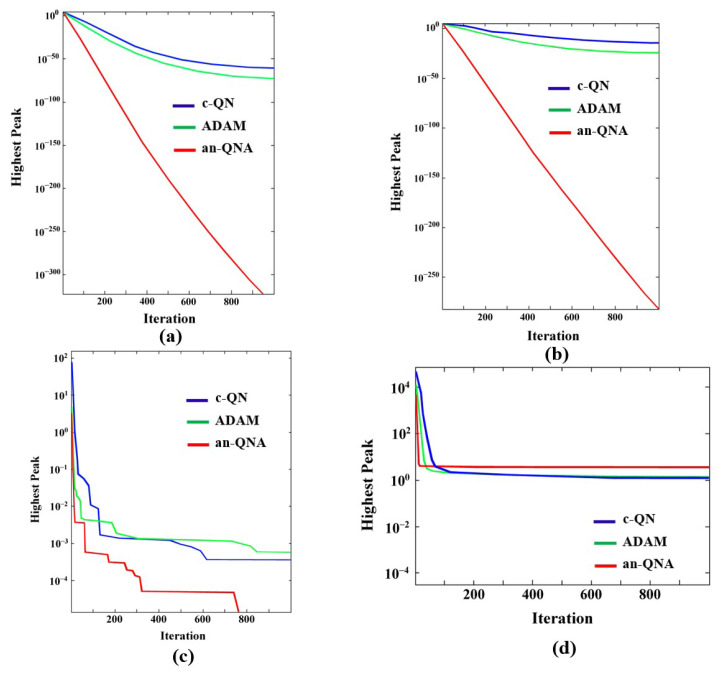
Convergence graph of unimodal benchmark functions (**a**) F_v1_ (**b**) F_v2_ (**c**) F_v3_ (**d**) F_v4_.

**Figure 7:**
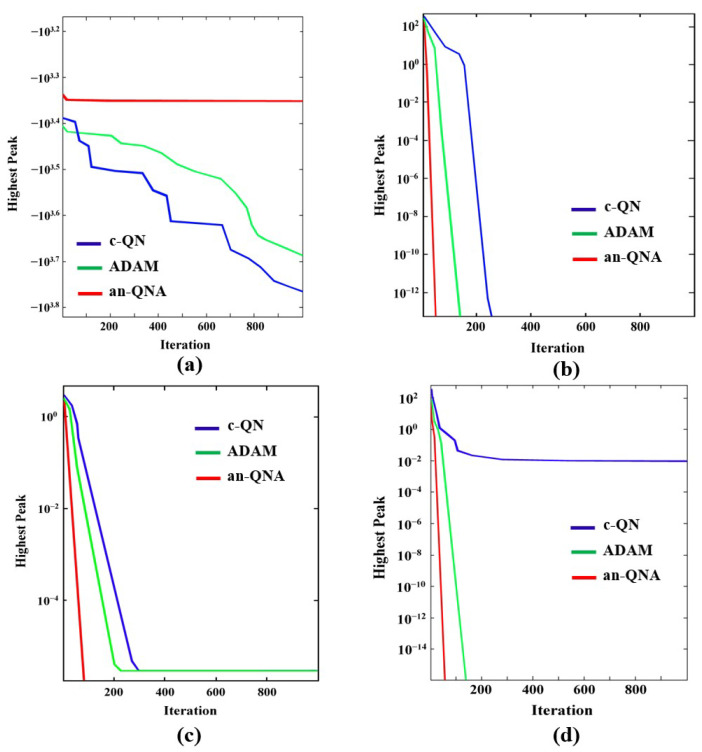
Convergence graph of multimodal benchmark functions (**a**) F_v5_ (**b**) F_v6_ (**c**) F_v7_ (**d**) F_v8_.

The c-QN, ADAM, and an-QNA algorithms were developed using MATLAB R2021a. Table 3, Table 4 and Table 5 indicate that the c-QN, ADAM, and an-QNA algorithms deliver the best optimal values for classical optimization problems in terms of the minimum and maximum function values compared to other OAs. Meanwhile, Table 6, Table 7 and Table 8 show that the an-QNA outperforms c-QN and ADAM in terms of producing higher-quality standard and mean values for most classical functions, yielding the smallest function values. Additionally, the convergence graphs in Figure 6 and Figure 7 demonstrate that an-QNA achieves the best possible optimal values for standard functions in fewer iterations than c-QN and ADAM. The results presented in Table 6, Table 7 and Table 8 clearly show that the proposed an-QNA variant surpasses the performance of other algorithms, including c-QN and ADAM, in terms of mean, standard deviation, and minimum/maximum cost function values while efficiently finding the optimum. Therefore, the an-QNA variant proves to be highly competitive with other OAs.

Furthermore, the convergence behaviors of c-QN, ADAM, and the proposed an-QNA algorithms have been analyzed, with the convergence curves depicted in Figure 6 and Figure 7. These curves reveal that the an-QNA consistently achieves better convergence points than the other algorithms. The obtained results confirm that the an-QNA is more effective in identifying the best optimal solution with fewer iterations. As a result, the an-QNA algorithm successfully avoids premature convergence to local optima and enhances the exploration of the search space.

## 3. Proposed an-QNA Based LNA Circuit

**Figure 8:**
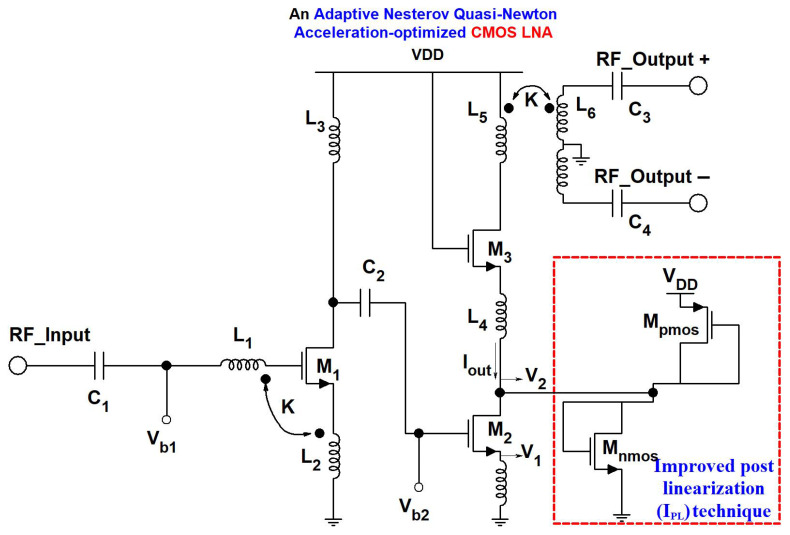
Proposed two-stage LNA using an-QNA.

The schematic of the proposed single-ended-to-differential LNA using an-QNA is shown in Figure 8. The designed LNA is composed of two stages. The first stage includes the transistor in a common-source configuration, and the second stage of the LNA includes a common-source transistor cascoded with a common-gate transistor. An improved post-linearization (I_PL_) technique is implemented to mitigate the non-linearity of the designed LNA. Table 9 shows the proposed LNA device component values using an-QNA.

An input matching of the designed LNA consists of a feedback transformer formed by the coupling of L_1_ and L_2_. The input impedance of the amplifier is approximately given by Equation (20):

(20) $$Z_{in}\approx \frac{1}{g_{m}}\frac{{\left(\frac{n}{k}\right)}^{2}}{\frac{n}{k}+1}$$

where g_m_ refers to the transconductance of the transistor M_1_,

(21) $$n=\sqrt{\frac{L_{1}}{L_{2}}}$$

**Figure 9:**
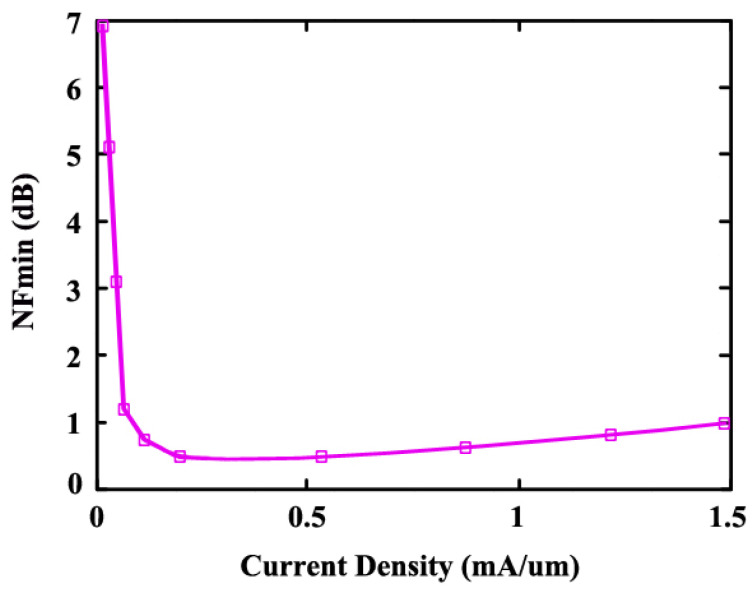
NF_min_ vs. current density.

Here, n is the turns ratio, and k is the coupling factor. The input matching is shown by Equation (21) and is frequency independent, which means ideally the input match is wideband. Thus, as the transformer layout has parasitic capacitances that depend on frequency and change over frequency, it limits the input-matching bandwidth. The use of a transformer-based matching technique is beneficial over traditional lumped components matching because it is wideband, and wideband is needed to meet the input return loss (S_11_) specification of −10 dB over the interested frequency band. The optimal sizing and biasing for the first-stage common-source transistor is selected for its minimal noise. Figure 9 shows the plot of the minimum noise NF_min_ versus the bias current of the M_1_ transistor of width 32 μm and gate length 65 nm. It shows that an NF_min_ of 0.5 dB at 0.5 mA can be achieved.

### Linearity Analysis

A list of distortion sources and linearization methods is presented in Table 10. From the table, we can determine that, in most reported linearization techniques, distortion of transconductance is suppressed at the 2nd and 3rd orders. In light of this, linearization of higher-order terms (beyond the third order) as well as output conductance remain open issues. Typically, the non-linearity in LNA arises because of the non-linear behavior of currents of transistors. In this work, to solve the non-linearity issue, rather than using the above-mentioned conventional technique, we have used a I_PL_ technique to increase the linearity.

In our circuit, the inductor L_4_ is connected between the drain and source of transistor M_2_, M_3_, and the parasitic capacitances at the source of M_3_ and at the drain M_2_ form a wideband π network. The proper sizing of L_4_ cancels the parasitic capacitive effects, which forms a short circuit over the whole frequency on interest. In this state, nonlinearity from transistor M_3_ can be ignored [34], which leaves the transistor M_2_ as the main source of non-linearity. The implemented I_PL_ technique facilitates partially canceling the second-order and third-order non-linearity, which can be analyzed by using the Taylor series expressions of the drain currents I_2_ of transistors M_2_, I_nmos_ of transistors M_nmos_, and I_pmos_ of transistors M_pmos_.

(22) $$i_{2}=g_{m1}v_{1}+g_{m1}v_{1}^{2}+g_{m3}v_{1}^{3}$$

(23) $$i_{nmos}=g_{mn1}v_{2}+g_{mn2}v_{2}^{2}+g_{mn3}v_{2}^{3}$$

(24) $$i_{pmos}=g_{mp1}v_{2}+g_{mp2}v_{2}^{2}+g_{mp3}v_{2}^{3}$$

(25) $$v_{2}=b_{1}v_{1}+b_{2}v_{1}^{2}+b_{3}v_{1}^{3}$$

where b_1_–b_3_ are, in general, frequency dependent. In practice, the π network cancels the effects of b_2_ and b_3_ at the frequency of interest formed by the inductor L_4_ and the parasitic capacitances at the drain of M_2_ and at the source M_3_.

(26) $$\begin{array}{c} i_{out}=i_{2}+i_{nmos}+i_{pmos}\\ =\left[g_{m1}-b_{1}\left(g_{mn1}+g_{mp1}\right)\right]v_{1}+\left[g_{m2}-b_{2}\left(g_{mn1}+g_{mp1}\right)\right]\\ +b_{1}^{2}\left(g_{mn2}+g_{mp2}\right)+\left[g_{m3}-b_{3}\left(g_{mn1}+g_{mp1}\right)+b_{1}^{3}\left(g_{mn3}+g_{mp3}\right)+2b_{1}b_{2}\left(g_{mn2}+g_{mp2}\right)\right]v_{1}^{3} \end{array}$$

**Figure 10:**
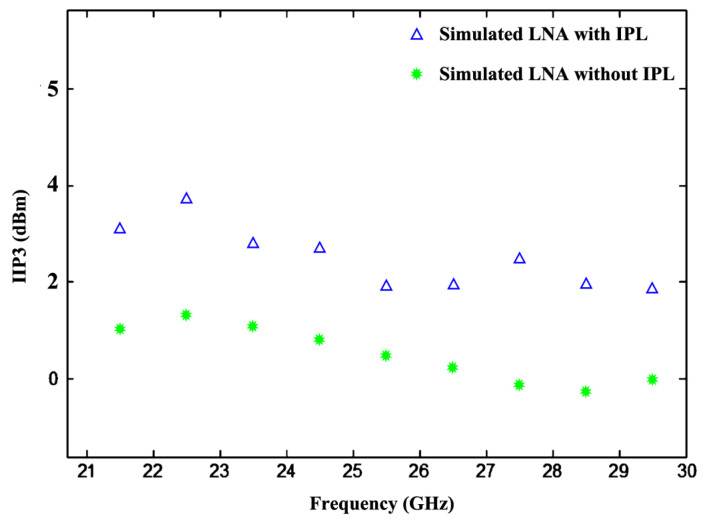
IIP3 vs. frequency.

The third-order non-linearity of the Iout (third term in (Equation (26))) should be zero or close to zero in order to attain a better IIP3. The I_PL_ technique uses voltage V_2_ and duplicates the non-linear drain current of transistor M_2_, partially canceling both second-order and third-order distortion terms. Finally, M_2_, M_n_, and M_p_ use the same finger sizes to improve matching and, hence, the cancellation of harmonics. The relationship between the M_2_ source voltage V_1_, M_2_ drain voltage V_2_, and the input voltage V_in_ at the gate of transistor M_2_ can be stated up to the third order in Equation (27), while the expression of IIP3 is calculated as shown in Equation (28):

(27) $$V_{1}=A_{1}\left(\omega \right)oV_{in}+A_{2}\left(\omega_{1}\omega_{2}oV_{in}^{2}+C_{3}\left(\omega_{1,}\omega_{2,}\omega_{3,}oV_{in}^{3}\right)\right)$$

(28) $$IIP3=20.log_{10}\left(\sqrt{|\frac{4}{3}.\frac{C_{1}\left(\omega \right)}{C_{3}\left(\omega_{1},\omega_{2},\omega_{3}\right)}}\right)+10\mathrm{dB}$$

where “o” is the Volterra series operator [35]. C_1_( ω ) is usually fixed by the design parameters; therefore, low distortion is achieved by reducing C_3_(ω_1_, ω_2_, ω_3_). The MOS transistor operated in saturation region exhibits third-order negative transconductance and second-order positive transconductance, so concurrently decreasing the second- and third-order transconductance increases IIP3. The simulated results of IIP3 vs. frequency with and without implemented I_PL_ are shown in Figure 10.

**Figure 11:**
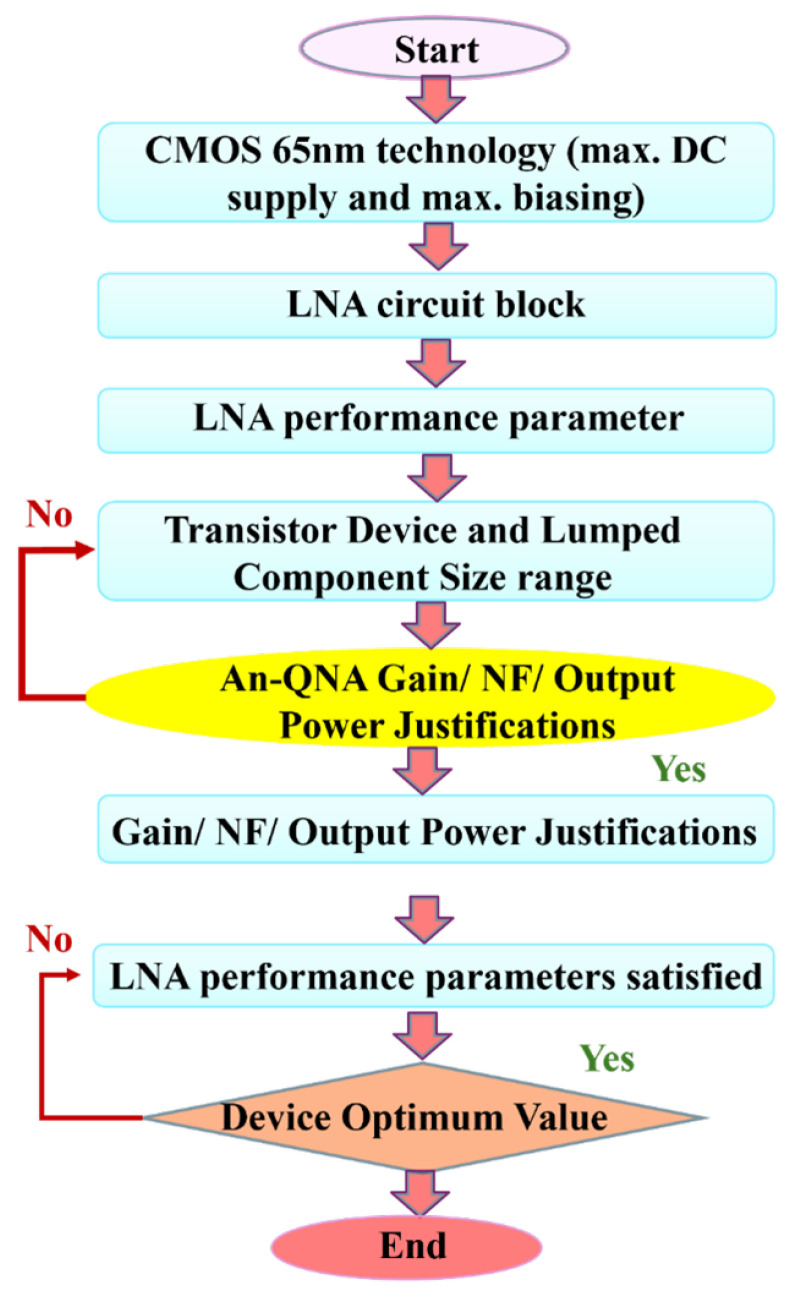
Flowchart of the proposed LNA using an-QNA.

Figure 11 depicts that the flowchart of the proposed LNA using the an-QNA algorithm begins with the design process initiation, where the optimized LNA circuit is developed using the an-QNA method. The first step is selecting the 65 nm CMOS technology, which serves as the basis for the LNA fabrication. Next, the single-ended-to-differential two-stage LNA circuit block is designed, establishing the core structure. Transistor devices and lumped components, such as resistors and capacitors, are then sized according to the performance needs. The an-QNA algorithm is applied to optimize key parameters like gain, NF, and output power, with modifications to guarantee global convergence through quadratic estimation. The performance of the LNA is subsequently evaluated, and if the specifications (such as gain, NF, and linearity) are met, then the design moves forward. If not, further optimization is performed. Finally, once all performance criteria are satisfied, the optimal values for the transistors and components are finalized, completing the design process of the LNA using the an-QNA method.

## 4. Results and Discussion

An LNA based on the proposed design is simulated on CMOS with 65 nm. The proposed an-QNA-based optimized LNA consumes 28 mW of power and operates at 1.8 volts.

**Figure 12:**
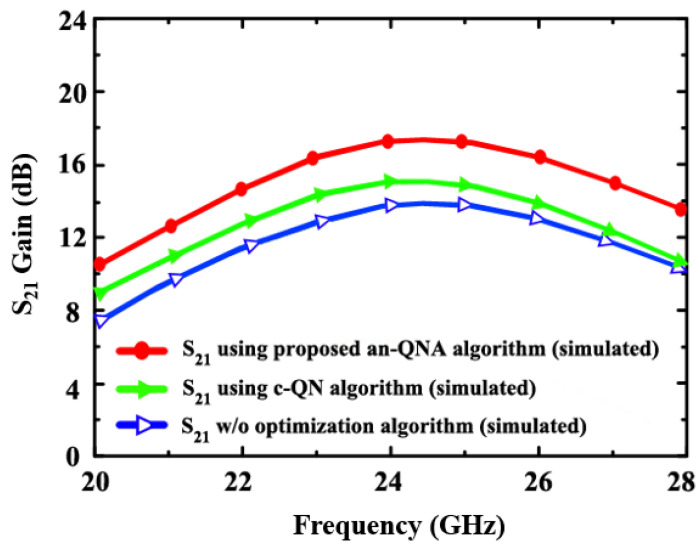
The simulated gain S_21_ of the proposed LNA (using an-QNA and c-QN and without optimization).

Figure 12 presents the simulated gain S_21_ of the proposed LNA (using an-QNA c-QN and, w/o optimization). In an-QNA, the simulated gain S_21_ is 17.5 dB, while in c-QN, it is 15 dB. A 13.9 dB simulated gain S_21_ without optimization is also achieved.

**Figure 13:**
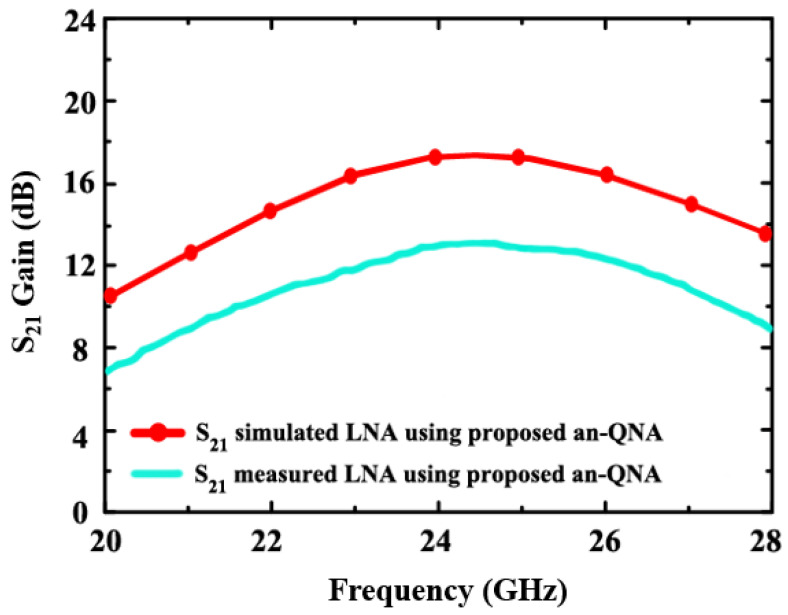
The simulated and measured gain S_21_ of the proposed LNA (using an-QNA).

In Figure 13, the simulated and measured gain S_21_ of the proposed LNA (using an-QNA) is shown. According to the simulation, the measured one is 12.9 dB, while the simulated one is 17.5 dB.

**Figure 14:**
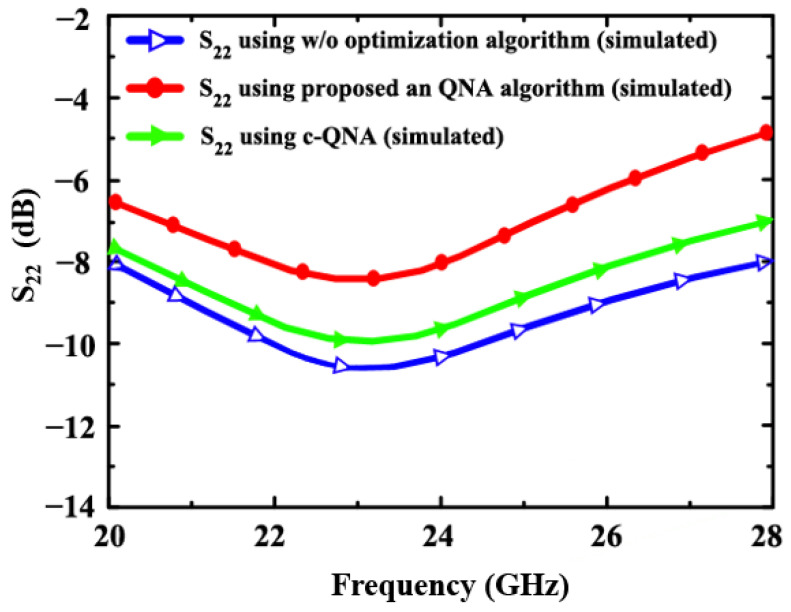
The simulated S_22_ of the proposed LNA (using an-QNA c-QN and, w/o optimization).

**Figure 15:**
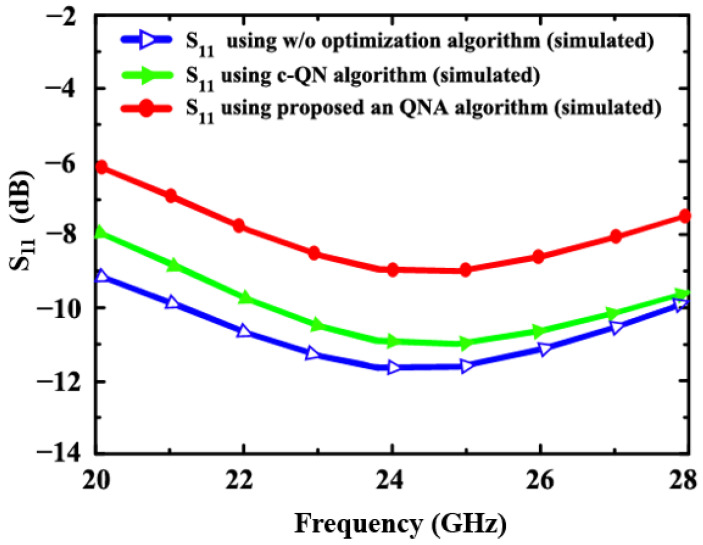
The simulated S_11_ of the proposed LNA (using an-QNA c-QN and, w/o optimization).

In Figure 14 and Figure 15, the simulated S_22_ and S_11_ for the proposed LNA (using an-QNA and c-QN and without optimization) is illustrated. The S_11_ simulation results are −9.1 dB, −10.9 dB, and −11.7 dB, and those for S_22_ (an-QNA and c-QN without optimization) are −8 dB, −9.8 dB, and −10.2 dB, respectively.

**Figure 16:**
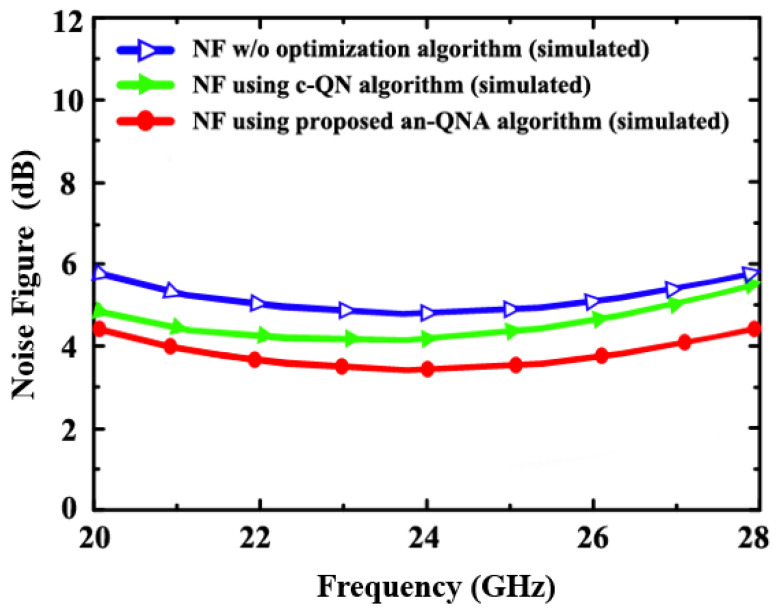
The simulated NF of the proposed LNA (using an-QNA and c-QN and without optimization).

**Figure 17:**
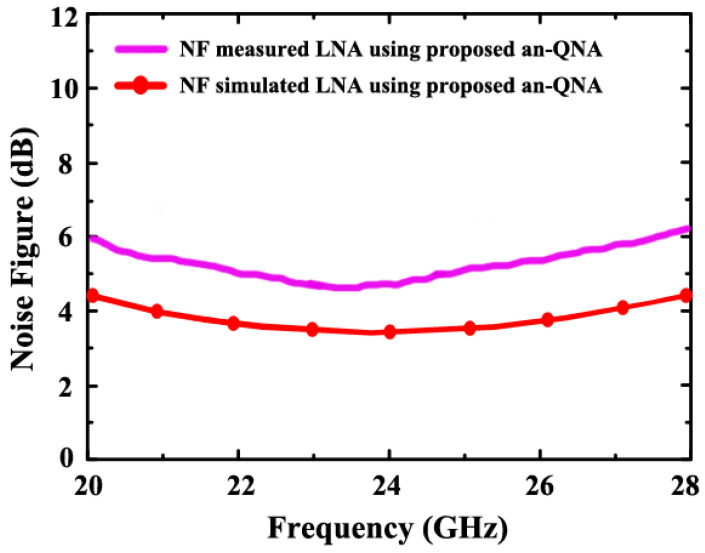
The simulated and measured NF (using an-QNA).

Figure 16 and Figure 17 demonstrate the simulated NF and measured NF of the proposed LNA (using an-QNA and c-QN and without optimization). The simulated NF is 3.7 dB, but the measured NF is 4.98 dB.

**Figure 18:**
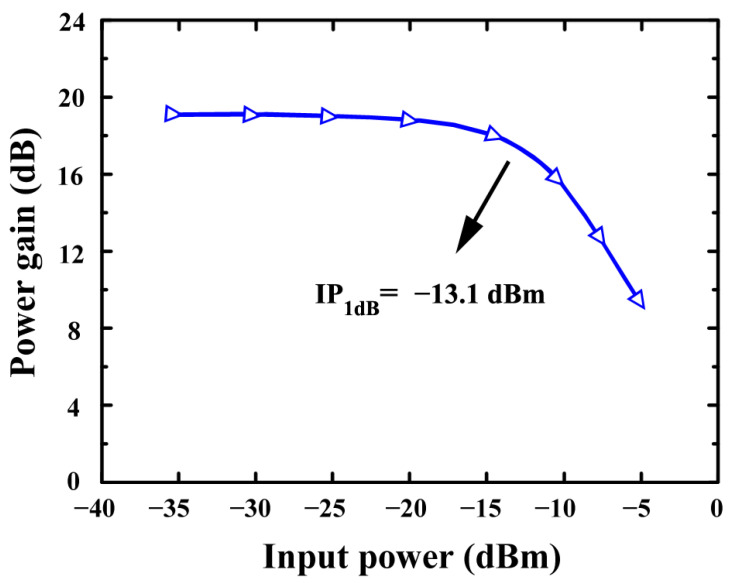
The simulated IP_1_dB of the proposed LNA (using an-QNA).

Figure 18 depicts the simulated IP_1_dB of the proposed LNA (using an-QNA). In simulations, the IP_1_dB of an LNA based on a QNA is achieved at −13.1 dB.

**Figure 19:**
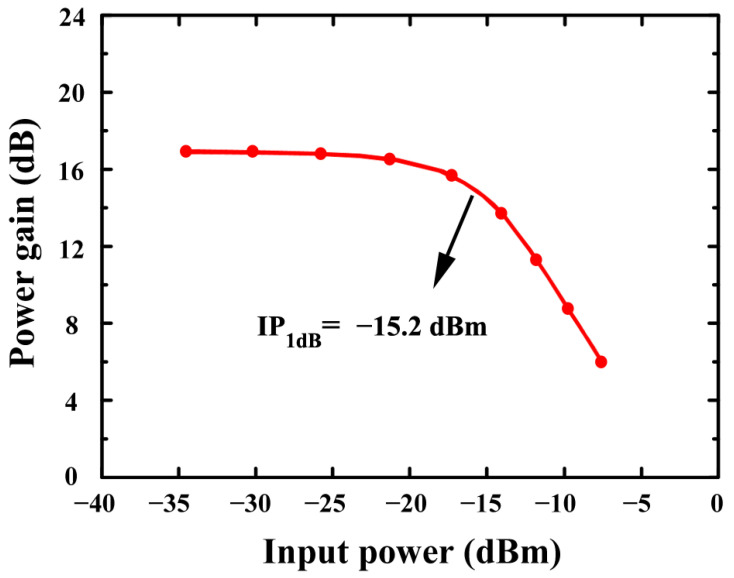
The simulated IP_1_dB of the proposed LNA (using c-QN)).

According to Figure 19, the proposed LNA’s IP_1_dB is simulated using c-QN. Based on simulations, an LNA using c-QN achieves −15.2 dB IP_1_dB.

**Figure 20:**
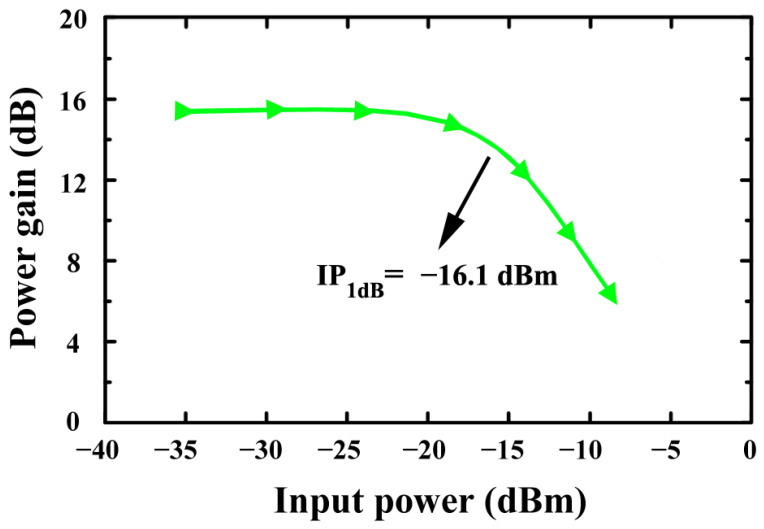
The simulated IP_1_dB of the proposed LNA (without optimization).

Figure 20 illustrates the simulated IP_1_dB of the proposed LNA (without optimization). The LNA without optimization has an IP_1_dB of −16.1 dB in simulations.

**Figure 21:**
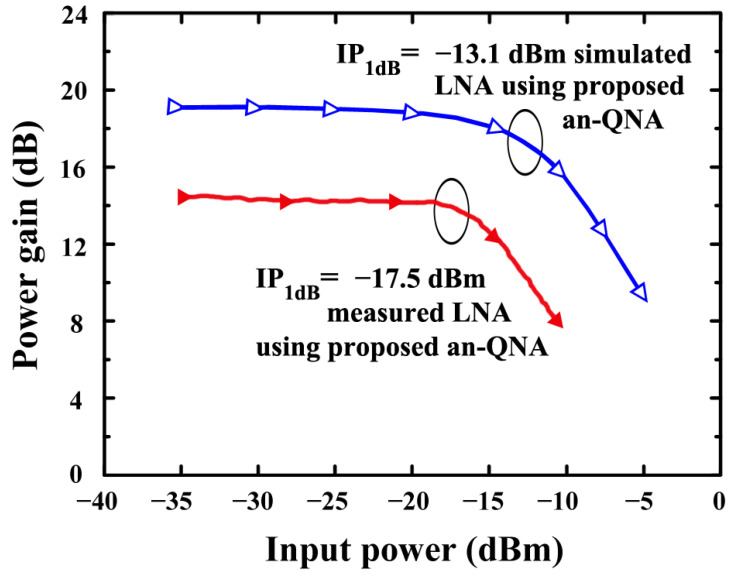
The measured and simulated IP_1_dB of the proposed LNA (using an-QNA).

Figure 21 depicts the simulated and measured IP_1_dB of the proposed LNA (using an-QNA). In simulations, the IP_1_dB of an LNA based on an-QNA is −13.1 dB, while measured one is −17.5 dB.

**Figure 22:**
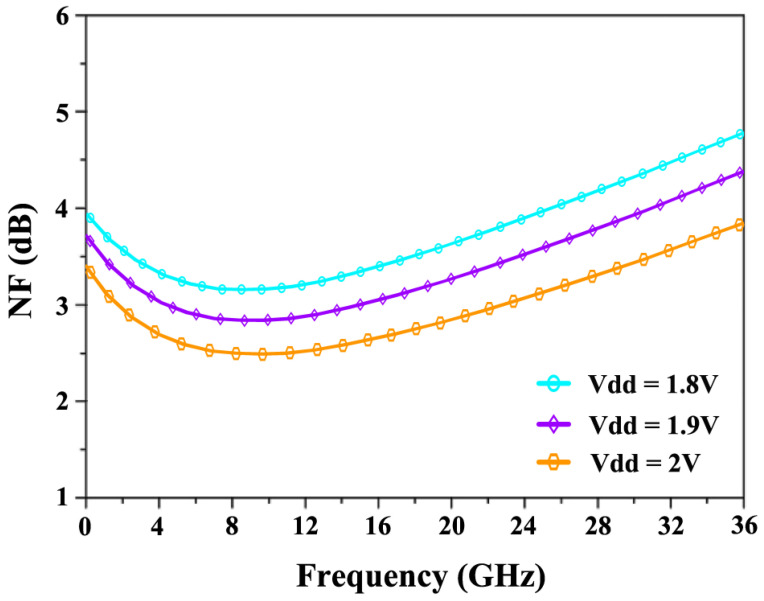
NF’s response to power supply variations.

**Figure 23:**
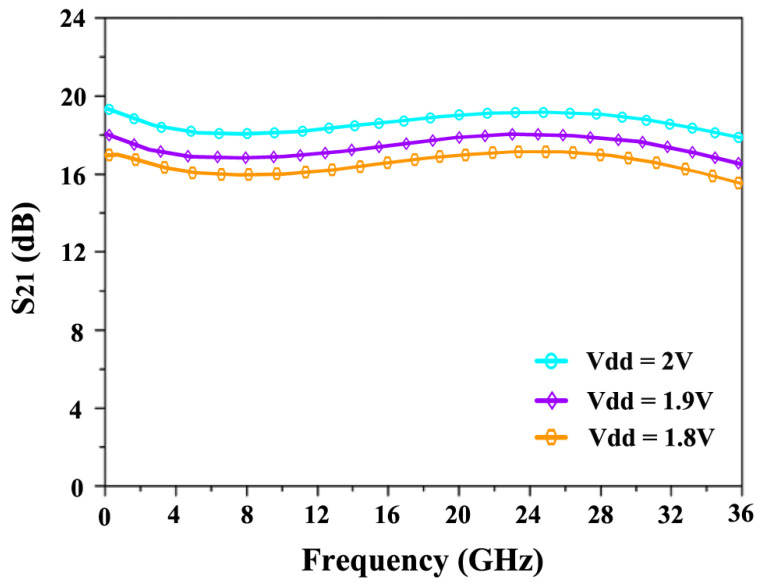
S_21_’s response to power supply variations.

Figure 22 illustrates the NF characteristics at different supply voltages (Vdd). The NF reaches a maximum value of 3.7 dB at 24 GHz when operating at 1.8 V, while it decreases to a minimum of 3 dB when the supply voltage is increased to 2.0 V. Figure 23 shows the variation in S_21_ across different voltages. At 1.8 V, the S_21_ achieves 17.5 dB, and as the voltage increases, S_21_ improves further, reaching 18 dB at 1.9 V and 18.9 dB at 2.0 V.

**Figure 24:**
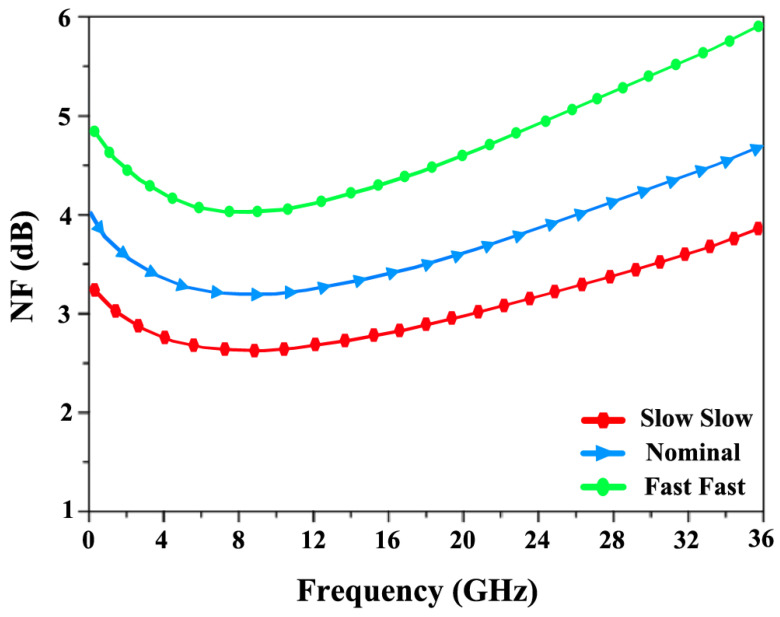
Corners simulations of nominal, slow–slow (SS), and fast–fast (FF) for NF.

**Figure 25:**
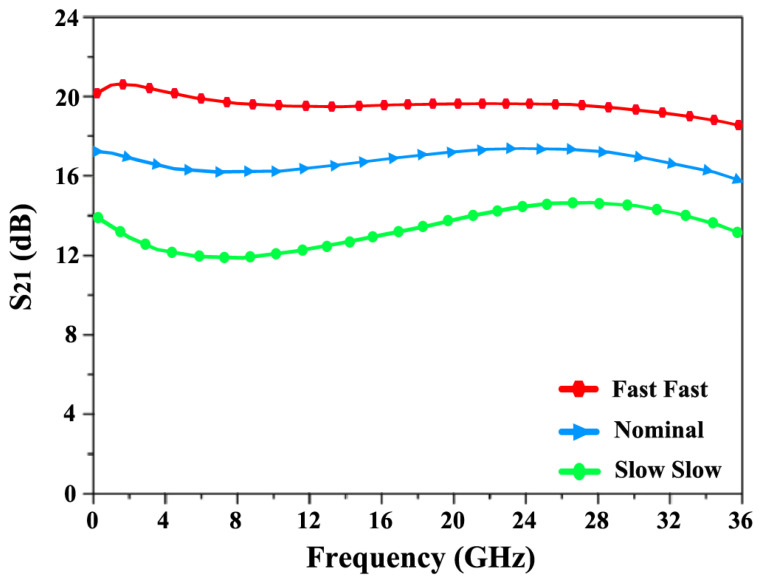
Corners simulations of nominal, slow–slow (SS), and fast–fast (FF) for S_21_.

To evaluate the variations from the nominal simulation results, a linear process corner analysis was conducted at the slow–slow (SS) and fast–fast (FF) corners in Figure 24 and Figure 25. It was observed that the NF fluctuates between 4.9 dB at the FF corner and 3.1 dB at the SS corner, with a nominal value of 3.7 dB. Meanwhile, the S_21_ reaches a minimum of 14.2 dB at the SS corner and a maximum of 19.8 dB at the FF corner, with a nominal value of 17.5 dB.

**Figure 26:**
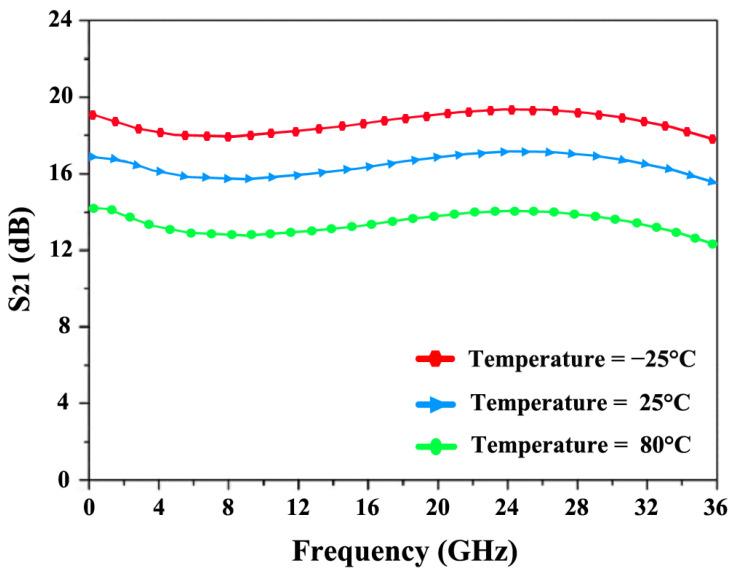
S_21_’s response to temperature variations.

**Figure 27:**
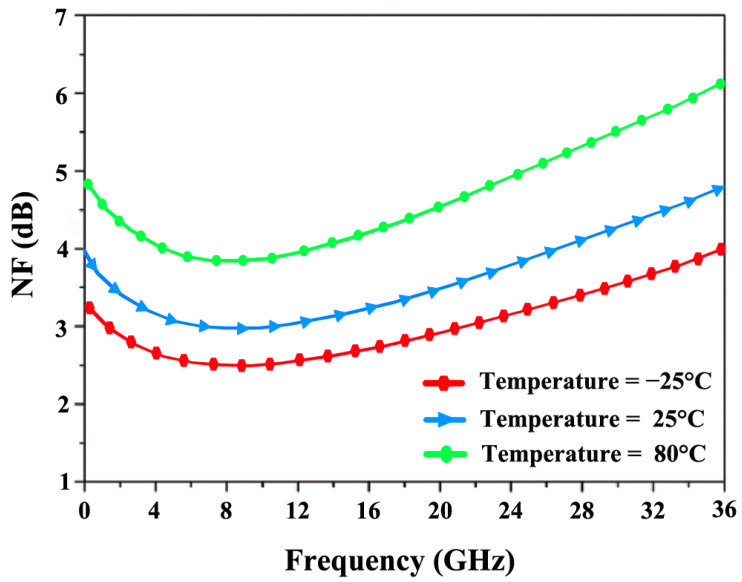
NF’s response to temperature variations.

Figure 26 explores the influence of temperature fluctuations, ranging from −25 °C to 80 °C, on the NF and power gain (S_21_) over the frequency range of 12 to 28 GHz. As the temperature rises, the NF increases, with a minimum of 4.9 dB at 80 °C and 3.1 dB at −25 °C. Additionally, the rise in temperature causes resistive components to consume more DC power, leading to a reduction in overall power gain. Figure 27 shows that S_21_ decreases from a maximum of 19.8 dB at −25 °C to a minimum of 14.2 dB at 80 °C.

**Figure 28:**
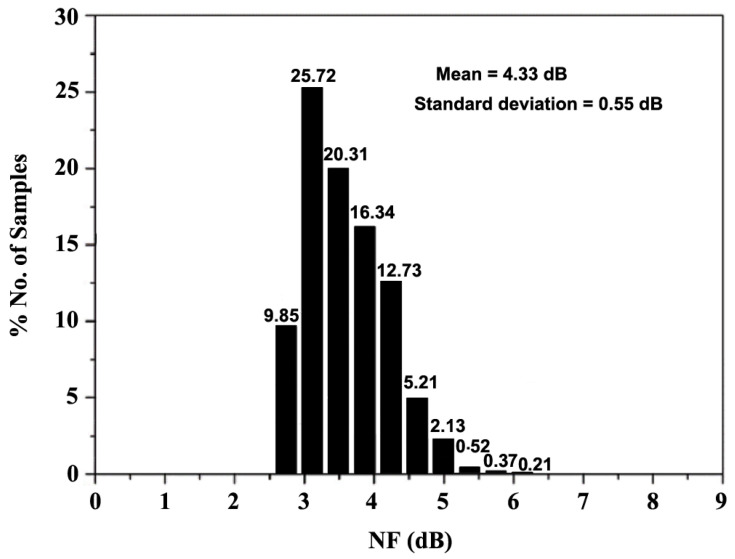
Monte Carlo analysis of NF.

**Figure 29:**
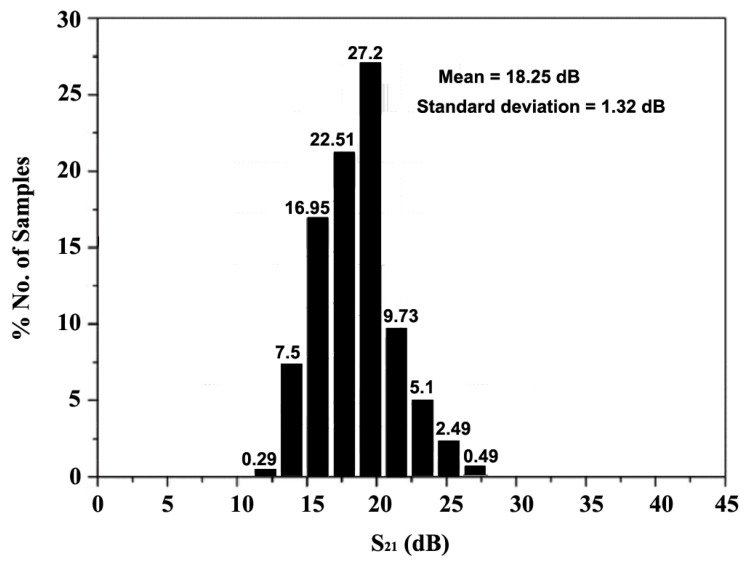
Monte Carlo analysis of S_21_.

The histogram for the NF, generated after 10,000 trials in the Monte Carlo (MC) analysis, provides a statistical distribution of the NF values. This analysis allows for the observation of how NF varies across the trials, offering insights into the overall performance and consistency of the system under varying conditions. Figure 28 and Figure 29 present the MC analysis, performed to assess the variations in NF and S_21_, respectively. For the frequency range of 10–30 GHz, 100 iterations were conducted to evaluate the frequency response of NF and S_21_, while the histogram plots of NF and S_21_ were generated after 10,000 trial runs. The MC analysis in Figure 28 reveals that 90.5% of the samples meet the requirements, with an NF below 3.7 dB after 10,000 trials. Additionally, Figure 29 shows that the mean NF is 4.33 dB with a standard deviation of 0.55 dB. As for S_21_, the results in Figure 29 indicate that the mean power gain is 18.25 dB with a standard deviation of 1.32 dB after 10,000 trials.

### Layout Issues

**Figure 30:**
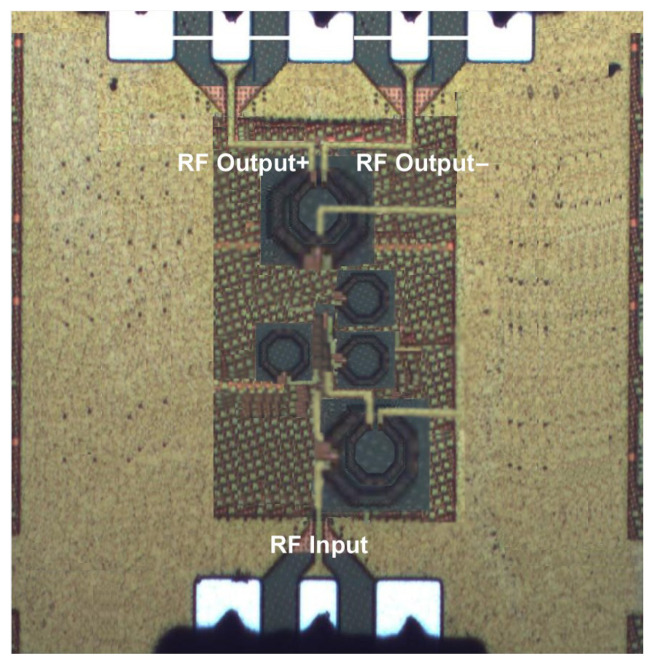
Chip photograph.

Figure 30 depicts the chip photograph of the proposed LNA. The proposed LNA has been designed and fabricated using CMOS 65 nm technology. The inductive and capacitive parasitic effects have been attempted to be minimized by carefully laid out the compact chip layout. To reduce the transistors’ series gate resistance and to avoid nonlinearity due to transistors’ parasitic shunt capacitance, the layout of LNA transistors was designed with a 2 μm width per finger. Two fF/μm^2^ metal–insulator–metal (MIM) capacitors were used in the LNA design, and a multilayer metal structure was used to minimize the inductance and resistance of the ground plane. The top metal layer, layer 9, was used to design the inductors with a quality factor (Q) of 13 at 24 GHz, and the output transformer was designed with a metal layer 8 and metal layer 9. The designed LNA chip die size was 0.67 mm^2^. The Cadence Spectre RF simulator was used to calculate the post-layout results of the designed LNA. The large-signal measurement of the LNA was achieved using the Agilent spectrum analyzer (PXA N9030 A) and Agilent signal generator (PSG 8257D). Small signal measurement was conducted by using the S parameter simulation technique, and it acquired specifications of LNA, which included gain, noise value, and reflection coefficient.

In Table 11, we summarize the performance characteristics of the other reported CMOS LNAs along with the proposed LNA.

## 5. Conclusions and Future Research

In conclusion, an-QNA method has been developed in this paper that significantly improves the performance of LNAs. Additionally, the proposed method accelerates convergence while improving linearity using I I_PL_ techniques and ensuring global convergence. A highly efficient LNA is designed to achieve enhanced performance using 65 nm CMOS technology, and as a result, achieves a simulated gain of 17.5 dB, an NF of 3.7 dB, as well as an (IP_1_dB) of −13.1 dBm. The optimized an-QNA-based LNA includes a measured gain of 12.9 dB, a measured NF of 4.98 dB, and a measured compression point of the input 1 dB of −17.8 dB. The chip area of the optimized LNA is 0.67 mm^2^. In automobile radar applications, these advancements demonstrate the potential of the an-QNA method. Future research could explore alternative CMOS nodes, such as 45 nm or 28 nm, that may enhance performance further. Moreover, further research is needed to optimize power consumption while maintaining performance, expand the frequency range, determine the robustness of the LNA against variations in the processing process, and integrate the LNA with other components of the radar system.

## Figures and Tables

**Table 1:** Summary of the simulation results of LNA modeling.

Name of Algorithm	Training_Error_ Median/Ave/Best/Worst (w) (×10^−3^)	Iteration Counts (k)	Time (s)	Test_Error_ Median/Ave/Best/Worst (w) (×10^−3^)	Rate of Convergence (%)
c-QN	0.75/0.76/0.62/0.91	41,828	95	0.640/0.901/0.534/2.01	75%
an-QNA	0.57/0.51/0.32/0.65	35,561	65	0.798/1.14/0.591/2.15	100%

**Table 2:** Multimodal and unimodal benchmark functions (dimensions, minimum range).

Function Name	Dimension	Range	Minimum Function (fmin)
Function_v1(unimodal) (y)_	30	[−100, 100]	0
Function_v2(unimodal) (y)_	30	[−100, 100]	0
Function_v3(unimodal) (y)_	30	[−100, 100]	0
Function_v4(unimodal) (y)_	30	[−1.28, 1.28]	0
Function_v5(multimodal) (y)_	30	[−500, 500]	−418.9 × 5
Function_v6(multimodal) (y)_	30	[−32, 32]	0
Function_v7(multimodal) (y)_	30	[−5.1, 5.1]	0
Function_v8(multimodal) (y)_	30	[−600, 600]	0

**Table 3:** The c-QN best solutions and statistical outcomes (unimodal benchmark functions).

c-QN	Minimum	Maximum	μ	α
	6.16 × 10^−61^	5.8 × 10^4^	215.8	2.5 × 10^3^
	1.97 × 10^−35^	1.43 × 10^12^	1.43 × 10^9^	4.52 × 10^10^
	3.3 × 10^−15^	1.8876 × 10^5^	1.7745 × 10^3^	1.0505 × 10^4^
	2.6647 × 10^−14^	89.07	2.61	12.13
	27.11	2.1621 × 10^8^	7.4518 × 10^5^	1.0897 × 10^7^
	1.2547	6.5240 × 10^4^	335.9	3.4849 × 10^3^
	3.61 × 10^−4^	92.21	0.49	5.80

**Table 4:** The ADAM best solutions and statistical outcomes (unimodal benchmark functions).

ADAM	Minimum	Maximum	μ	α
	2.71 × 10^−73^	6.15 × 10^4^	173.1716	2.53 × 10^3^
	7.42 × 10^−43^	3.2669 × 10^9^	3.2708 × 10^6^	1.03 × 10^8^
	2.52 × 10^−25^	1.1651 × 10^5^	674.7361	5.80 × 10^3^
	2.42 × 10^−20^	88.03	0.945	6.765
	27.16	2.30 × 10^8^	6.71 × 10^5^	9.98 × 10^6^
	1.25	7.07 × 10^4^	197.70	2.81 × 10^3^
	5.791 × 10^−4^	80.65	0.133	2.668

**Table 5:** The an-QNA best solutions and statistical outcomes (unimodal benchmark functions).

an-QNA	Minimum	Maximum	μ	α
	0.00	7.10 × 10^4^	125.5	2.4 × 10^3^
	1.60 × 10^−175^	3.78 × 10^13^	1.27 × 10^10^	1.1956 × 10^12^
	1.2 × 10^−282^	6.174 × 10^4^	1.2536 × 10^3^	1.0787 × 10^4^
	1.7 × 10^−152^	88.03	0.349	4.369
	27.10	2.30 × 10^8^	3.5409 × 10^5^	1.00 × 10^7^
	3.87	7.07 × 10^4^	123.81	2.5129 × 10^3^
	1.41 × 10^−5^	80.652	0.202	4.97

**Table 6:** The c-QN best solutions and statistical outcomes (multimodal benchmark functions).

c-QN	Minimum	Maximum	μ	α
	−5.80 × 10^3^	−2.44 × 10^3^	−4.03 × 10^3^	967.79
	5.68 × 10^−14^	458.78	10.78	48.011
	1.50 × 10^−14^	20.7623	0.412	2.276
	0.009	665.77	3.149	32.93
	0.03	5.520 × 10^8^	1.57 × 10^6^	2.49 × 10^7^
	0.69	8.056 × 10^8^	3.20 × 10^6^	4.36 × 10^7^

**Table 7:** The ADAM best solutions and statistical outcomes (multimodal benchmark functions).

ADAM	Minimum	Maximum	μ	α
	−4.8344 × 10^3^	−2.5399 × 10^3^	−3.3352 × 10^3^	731.9012
	0	438.1148	4.9465	32.6783
	1.5099 × 10^−14^	20.7623	0.2497	1.7307
	0	527.3462	1.4174	20.4885
	0.0538	6.1414 × 10^8^	1.1003 × 10^6^	2.1982 × 10^7^
	1.11284	9.0172 × 10^8^	1.5261 × 10^6^	3.2837 × 10^7^

**Table 8:** The an-QNA best solutions and statistical outcomes (multimodal benchmark functions).

an-QNA	Minimum	Maximum	μ	α
	−2.25 × 10^3^	−2.23 × 10^3^	−2.25 × 10^3^	6.3372
	0	488.07	2.33	26.43
	1.4 × 10^−15^	20.8472	0.1045	1.1726
	0	555.03	0.959	18.93
	0.558	8.105 × 10^8^	1.04 × 10^6^	2.15 × 10^7^
	0.193	9.23 × 10^8^	1.103 × 10^6^	2.9672 × 10^7^

**Table 9:** Proposed LNA device component values using an-QNA.

Element	Dimension
M_1_	32 µm/65 nm
M_2_	54 µm/65 nm
M_3_	54 µm/65 nm
C_1_, C_2_, C_3_	1 pF, 4.2 pF, 1.2 pF
L1∼L_3_	1.3 nH, 1.5 nH, 170 pH
L4∼L_6_	1.5 nH, 1.8 nH, 154 pH

**Table 10:** An description of distortion sources and linearization methods [33].

Distortion Sources	g_m_				g_ds_
Methods of Linearization	Intrinsic 2nd Order	Intrinsic 3rd Order	2nd-Order Interaction	Higher Order	
Feedback	✓	✓		✓	
Harmonic termination		✓	✓		
Optimal biasing		✓			
Feedforward	✓	✓		✓	
Derivative superposition (DS)		✓			
Complementary DS	✓	✓			
Differential DS	✓	✓			
Modified DS		✓	✓		
IM2 injection		✓	✓		
Noise/distortion cancellation	✓	✓			✓
Post-Distortion	✓	✓			

**Table 11:** Performance characteristics of other reported CMOS LNAs.

Ref.	Tech (nm)	V_DD_ (V)	FrEquation (GHz)	S_21_ (dB)	NF (dB)	IP_1_dB	Power (mW)
[36]	65 nm	1.5	60	11.289	1.819	N/A	7.25
[37]	180 nm	N/A	24	12.8	3.3	N/A	8
[38]	0.25 μm BiCMOS	1.8	16–43	10.5	2.5-4	1.8 (IIP3)	24
[39]	0.13 μm CMOS	12	27–31	22.14	1.86	−16 (IIP3)	33.4
[40]	45 nm	N/A	5.0–27.5	18.57 (PSO)	2.4–3.1	N/A	1.6
[41]	180 nm	1.8	5.5	22.15 (firefly)	1.16	−2.60 (IIP3)	N/A
[This Work]	65 nm	1.8	24	17.5/12.9 (sim./meas) (an-QNA)	3.7/4.98 (sim./meas) (an-QNA)	−13.1/−17.8 (sim./meas) (an-QNA)	28

## Data Availability

Data are contained within the article.
